# Simple Preparation of Pacific Cod Trypsin for Enzymatic Peptide Synthesis

**DOI:** 10.4061/2011/912382

**Published:** 2011-09-19

**Authors:** Tomoyoshi Fuchise, Haruo Sekizaki, Hideki Kishimura, Sappasith Klomklao, Sitthipong Nalinanon, Soottawat Benjakul, Byung-Soo Chun

**Affiliations:** ^1^Faculty of Pharmaceutical Sciences, Health Sciences University of Hokkaido, Ishikari-Tobetsu, Hokkaido 061-0293, Japan; ^2^Laboratory of Marine Products and Food Science, Research Faculty of Fisheries Sciences, Hokkaido University, Hakodate, Hokkaido 041-8611, Japan; ^3^Department of Food Science and Technology, Faculty of Technology and Community Development, Thaksin University, Phattalung Campus, Phattalung 93110, Thailand; ^4^Faculty of Agro-Industry, King Mongkut's Institute of Technology Ladkrabang, Choakhunthaharn Building, Choakhunthaharn Road, Ladkrabang, Bangkok 10520, Thailand; ^5^Department of Food Technology, Faculty of Agro-Industry, Prince of Songkla University, Hat Yai, Songkhla 90112, Thailand; ^6^Faculty of Food Science and Biotechnology, Pukyong National University, Busan 608-737, Republic of Korea

## Abstract

Trypsin from the pyloric caeca of Pacific cod (*Gadus macrocephalus*) was easily prepared by affinity chromatography on Benzamidine Sepharose 6B and gel filtration on Superdex 75. Pacific cod trypsin was composed of three isozymes, and their molecular masses were estimated 23,756.34 Da, 23,939.62 Da, and 24,114.81 Da by desorption/ionization time-of-flight mass spectroscopy (MALDI/TOF-MS) and their isoelectric points (pIs) were approximately 5.1, 6.0, and 6.2, respectively. The isolated Pacific cod trypsin showed high similarity to other frigid-zone fish trypsins. The kinetic behavior of tryptic hydrolysis toward *N*-*p*-tosyl-L-arginine methyl ester hydrochloride (TAME), *N*-benzoyl-L-arginine *p*-nitroanilide hydrochloride (BAPA), and *p*-amidinophenyl ester were also analyzed. In addition, the cod trypsin-catalyzed dipeptide synthesis was investigated using twelve series of “inverse subdtrates” that is *p*- and *m*-isomer of amidinophenyl, guanidinophenyl, (amidinomethyl)phenyl, (guanidinomethyl)phenyl, and four position isomers of guanidinonaphtyl esters derived from *N*-(*tert*-butoxycarbonyl)amino acid as acyl donor components. They were found to couple with an acyl acceptor such as L-alanine *p*-nitroanilide to produce dipeptide in the presence of the trypsin. All inverse substrates tested in this study undergo less enantioselective coupling reaction. The *p*-guanidinophenyl ester was most practical substrate in twelve series tested. The enzymatic hydrolysis of the resulting products was negligible.

## 1. Introduction

Proteinases from cold-water fish are of interest to us owing to their greater proteolytic activity towards native protein substrates and lower activation energy for catalysis compared with proteinases from mammalian or microbial sources [1]. Also, their survival in cold water required adaptation of their proteinase activity to low temperatures of their habitats. Proteinases from cold-adapted fish thus often have higher enzymatic activity at low temperatures than those from warm-blooded animals [2, 3]. High activity of these fish proteinases at low temperatures may be interesting for several industrial applications, such as in certain food processing operations that require low processing temperatures. Furthermore, proteinases from cold adapted fish inactivated at relatively low temperatures making such enzymes potentially useful in food applications where easy enzyme denaturation is desirable [4]. One of the main digestive proteinases, which are detected in pyloric caeca and intestine of fish, is trypsin (EC 3.4.21.4). Trypsin is a member of a large family of serine proteinases and cleaves the peptide bond on the carboxyl side of arginine and lysine. Recently, we isolated trypsins from the pyloric caeca of Pacific cod (*Gadus macrocephalus*) and the trypsin showed a lower optimum temperature and heat stability than those of temperate-zone fish, tropical-zone fish, and mammalian trypsins [5]. The characteristics suggested that the viscera of frigid-zone fish would be a potential source of trypsin for food processing operations.

**Figure 1:**
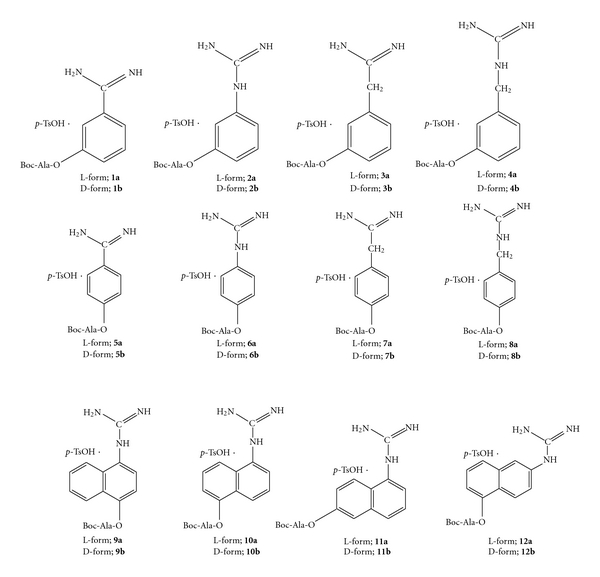
Structure of inverse substrates.

On the other hand, peptide synthesis using protease-catalyzed reverse reaction has been extensively studied with a variety of amino acids and peptide derivatives as coupling components [6–12]. It has been reported that the protease-catalyzed peptide synthesis is superior compared to the chemical coupling methods due to the requirement of less side chain protection. The method, however, has not been fully exploited for the possible synthesis of a number of biologically important peptides containing D-amino acid or other unnatural amino acid, because the enzymatic method is subject to restriction by substrate specificity and stereoselectivity. Previously, we reported that *p*-amidinophenyl esters [13] and *p*-guanidinophenyl esters [14, 15] behave as specific substrates for trypsin and trypsin-like enzymes. In these esters, the site-specific groups (charged amidinium and guanidinium) for the enzyme are included in the leaving-group portion instead of being in the acyl moiety. Such substrates are termed an inverse substrate (Figure 1) [16]. The inverse substrates allow the specific introduction of an acyl group carrying a nonspecific residue into the enzyme active site. The characteristic features of inverse substrates suggested that they are useful for enzymatic peptide synthesis. We demonstrated the successful application of inverse substrates for trypsin-catalyzed coupling [13, 17]. Recently, Bordusa [11] reported an enzymatic coupling reaction using inverse substrates and a wide variety of proteases, such as thrombin, clostripain, V8 protease, chymotrypsin and subtilisin. We also reported that the substrate specificity and yield were different by origin of trypsin [18, 19].

**Scheme 1:**
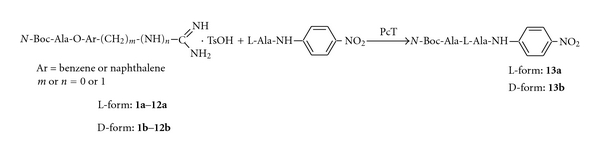
Enzymatic peptide synthesis using inverse substrate.

**Figure 2:**
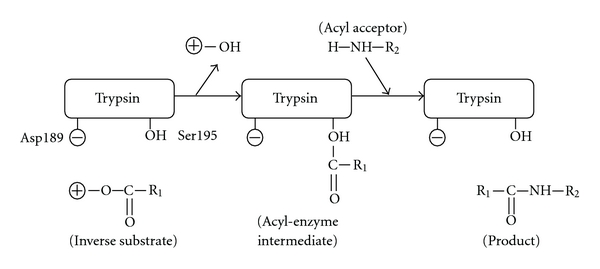
Reaction process of trypsin-catalyzed peptide bond formation with inverse substrate.

In the present paper, we describe the simple separation of trypsin from the pyloric caeca of Pacific cod by affinity chromatography for enzymatic peptide synthesis using inverse substrates (Scheme 1, Figure 2). Also, we report the selection of practical inverse substrates for Pacific cod trypsin-catalyzed peptide synthesis.

## 2. Materials and Methods

### 2.1. Materials

Pacific cod (*Gadus macrocephalus*) was caught off Hokkaido Prefecture, Japan. The pyloric caeca of Pacific cod were separated from other intestinal organs and were stored at −84°C prior to homogenization.

*N*-*p*-Tosyl-L-arginine methyl ester hydrochloride (TAME), *N*-benzoyl-L-arginine *p*-nitroanilide hydrochlodide (BAPA), *p*-nitrophenyl-*p *′-guanidinobenzoate hydrochloride (NPGB), tris(hydroxymethyl)aminomethane (Tris), and 3,5-dimethoxy-4-hydroxycinnamic acid (sinapinic acid) ware obtained from Sigma-Aldrich (Tokyo, Japan). *p*-Nitrophenol, HPLC grade dimethyl sulfoxide (DMSO), acetone, and acetonitril were purchased from Kanto Chemical Co. Inc. (Tokyo, Japan). L-Alanine *p*-nitroanilide (L-Ala-*p*NA) was purchased from Peptide Institute, Inc (Osaka, Japan). 3-(*N*-Morpholino)propanesulfonic acid (MOPS) was obtained from Wako Pure Chemicals (Osaka, Japan). The inverse substrate was prepared according to our previous paper [13, 15, 20–22]. Benzamidine Sepharose 6B and Superdex 75 were purchased from GE Healthcare (Buckinghamshire, England). Microplate reader was purchased from Tecan (Kanagawa, Japan). F96 microwell plate and UV plate were purchased from Nunc (Buckinghamshire, England) and Corning (Tokyo, Japan), respectively.

### 2.2. Preparation of Pacific Cod Trypsin

The frozen pyloric caeca were homogenized in three volumes of cold acetone (−20°C) using a homogenizer (*AM*-8; Nihonseiki Kaisha Ltd., Tokyo, Japan) for 3 min, and the homogenate was filtrated *in vacuo* on ADVANTEC No. 2 filter paper. Similarly the residue was homogenized in one volume of cold acetone, and was done twice more at same. The residue was air-dried at 4°C, and was stored at 4°C prior to enzyme extraction. Trypsin preparation steps below were carried out at 4°C.

The defatted powder (40 g) was mixed with 400 mL of buffer A [50 mM Tris-HCl buffer (pH 7.8) containing 0.5 M NaCl and 20 mM CaCl_2_]. A trypsinogen in the slurry was activated by incubation for 46 hr at 4°C. The slurry was centrifuged at 11,500 rpm for 30 min. The supernatant was pooled, and the other precipitate was extracted again with 200 mL of buffer A. The collected supernatant was subjected to solid (NH_4_)_2_SO_4_ fractionation and the precipitate in the 80% saturation was collected by centrifugation. The resultant precipitate was then dissolved in 500 mL of buffer A prior to centrifugation. The supernatant was collected and referred to as “crude enzyme.” The crude enzyme was applied to a Benzamidine Sepharose 6B affinity column (i.d. 2.5 × 6 cm) preequilibrated with buffer A. The column was washed with buffer A until no further decrease in absorbance at 280 nm was observed. The main trypsin fraction was eluted with buffer A containing 20 mM benzamidine. The fraction was concentrated on Amicon-stirred cell (YM10 ultrafiltration membrane, Tokyo, Japan) and applied to Superdex 75 column (i.d. 2.6 × 100 cm) preequilibrated with buffer A using the FPLC system (GE Healthcare). The peak containing TAME esterase activity was collected each and was concentrated on Amicon-stirred cell. The concentrate (about 5 mL) was dialyzed against 1 L of 50 mM NH_4_HCO_3 _  solution for 1 hr. The dialysate (purified Pacific cod trypsin) was lyophilized and was stored at −20°C until used in subsequent studies.

### 2.3. Protein Determination and Assay for Trypsin Activity

Protein concentrations were determined using a protein assay kit (BIO-RAD) with bovine serum albumin as the standard protein [23].

Trypsin activity was measured by the method of Hummel [24] using TAME as a substrate at 30°C to compare the enzymatic properties with previous our studies [5]. One unit of enzyme activity was defined as the amount of the enzyme hydrolyzing one micromole of TAME in a minute. The pH dependencies of trypsin were determined in 50 mM buffer solutions [acetic acid-sodium acetate (pH 4.0–7.0), Tris-HCl (pH 7.0–9.0), and glycine-NaOH (pH 9.0–11.0)] at 30°C. The temperature dependencies of trypsin were determined at pH 8.0 and at various temperatures. The temperature and pH stabilities of trypsin were found by incubating enzyme at pH 8.0 for 15 min at a range of 20–80°C and by incubating the enzyme at 30°C for 30 min at a range of pH 4.0–11.0, respectively.

### 2.4. Kinetic Measurements

First, the crystallized *p*-nitrophenol was utilized to determine the path length of the solution volume used in the microplate wells during the assay according to reported procedure [25]. The concentration of active sites of trypsins was determined with NPGB from the “burst” of *p*-nitrophenol at 405 nm (extinction coefficient = 18,200 M^−1^cm^−1^), following the method of Chase and Shaw [26]. 

Amidase activity as determined by using BAPA as a substrate according to the method of Erlanger et al. [27]. The amount of *p*-nitroaniline liberated from BAPA was determined by the increase in absorbance at 405 nm (extinction coefficient = 10,200 M^−1^cm^−1^). Esterase activity was determined by using TAME as a substrate according to the method of Hummel [24], measuring the change in absorbance at 247 nm (extinction coefficient = 540 M^−1^cm^−1^). Kinetic parameters using *N*-(*tert*-butoxycarbonyl)-L-alanine *p*-amidinophenyl esters (*N*-Boc-L-Ala-O*p*Am, **5a**) as inverse substrate were determined as previously described [16]. Changes of optical density were monitored at 305 nm (extinction coefficient = 16,700 M^−1^cm^−1^). Optical density changes at 305 nm (extinction coefficient = 16,700 M^−1^cm^−1^) were monitored.

The final reaction mixture (200 *μ*L) consisted of 180 *μ*L of 50 mM Tris-HCl (pH 8.0) containing 10 mM CaCl_2_, 10 *μ*L of substrate in DMSO, and 10 *μ*L of PcT solution in 10 mM Tris-HCl (pH 8.0) containing 2 mM CaCl_2_. Kinetics parameters were determined at eight substrate concentrations in the range of 0.01–0.08 mM for BAPA, 0.004–0.039 mM for TAME, and 0.0003–0.0038 mM for *N*-Boc-L-Ala-O*p*Am (**5a**) at 30°C. Kinetic constants were obtained by least-squares fitting of initial velocity data to Lineweaver-Burk transformation of the Michaelis-Menten equation.

### 2.5. Polyacrylamide Gel Electrophoresis

Sodium dodecyl sulfate-polyacrylamide gel electrophoresis (SDS-PAGE) was carried out using a 0.1% SDS-12.5% polyacrylamide slab-gel by the method of Laemmli [28]. The gel was stained with 0.1% Coomassie Brilliant Blue R-250 (CBB) in 50% methanol-7% acetic acid and the background of the gel was destained with 7% acetic acid.

Isoelectric focusing (IEF) was carried out with a 111 Mini IEF Cell system (BIO-RAD) according to attached manual. The gel was fixed with 4% sulfosalicylic acid-12.5% trichloroacetic acid-30% methanol and was stained with 0.5% CuSO_4_-10% acetic acid-27% ethanol-0.04% CBB. The background of the gel was destained with 0.5% CuSO_4_-7% acetic acid-12% ethanol and then 7% acetic acid-24% ethanol.

### 2.6. Determination of Molecular Mass

Pacific cod trypsin was analyzed by matrix-assisted desorption/ionization time-of-flight mass spectroscopy (MALDI/TOF-MS) using a Voyager PK2 (Applied Biosystems). The matrix (10 mg/mL) was prepared by dissolving sinapinic acid in 7 : 3 (acetonitrile: 0.1% TFA, 1 : 1, v/v), and used the supernatant.

### 2.7. Analysis of Amino Acid Sequence

To analyze the *N*-terminal sequence, the purified enzyme was electroblotted on to polyvinylidene difluoride (PVDF) membrane (Immobilon-P, Millipore) after SDS-PAGE. The amino acid sequence of the enzyme was analyzed by using a protein sequencer, Procise 492 (Perkin Elmer, Foster City, Califf, USA).

### 2.8. Pacific Cod Trypsin-Catalyzed Dipeptide Coupling Reaction

A solution of 40 *μ*L of 50 mM MOPS buffer (containing 20 mM of CaCl_2_, pH 8.0), 30 *μ*L of DMSO, 10 *μ*L of acyl acceptor (200 mM L-Ala-*p*NA in DMSO), and 10 *μ*L of trypsin solution (100 *μ*M, in 10 mM Tris buffer (pH 8.0) containing 2 mM CaCl_2_) were mixed. To this mixture, 10 *μ*L of acyl donor (10 mM inverse substrate in DMSO) was added and incubated at 25°C. The progress of the coupling reaction was monitored by HPLC under the following conditions: Shim-pack CLC-ODS (M) (column i.d. 4.6 × 250 mm), isocratic elution at 1 mL/min, 50% acetonitrile containing 0.1% trifluoroacetic acid. An aliquot of the reaction mixture was injected and the eluate was monitored at 310 nm (chromophore due to the *p*-nitroanilide moiety). Peak identification was made by correlating the retention time with that of authentic samples which were chemically synthesized [17]. Observed peak areas were used for the estimation of sample concentration.

## 3. Results and Discussion

### 3.1. Simple Preparation of Pacific Cod Trypsin

**Figure 3:**
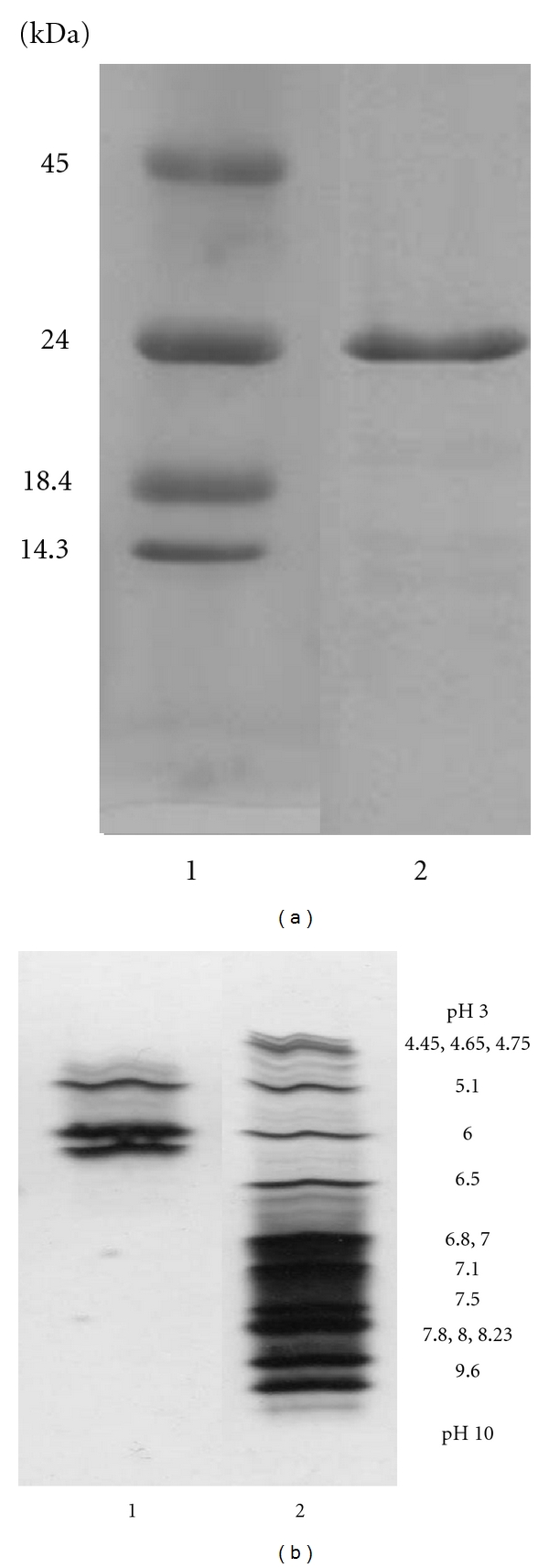
SDS-PAGE and IEF of purified Pacific cod trypsin. (a) SDS-PAGE of purified Pacific cod trypsin. Lane 1 contains protein standards; egg albumin (molecular weight, 45.0 kDa), bovine pancreatic trypsinogen (24.0 kDa), bovine milk *β*-lactoglobulin (18.4 kDa), and egg white lysozyme (14.3 kDa). (b) IEF of purified Pacific cod trypsin. Lane 1 contains purified Pacific cod trypsin. Lane 2 contains IEF isoelectric point (pI) standards: phycocyanin (pI 4.45, 4.65, 4.75), *β*-lactoglobulin (5.1), bovine carbonic anhydrase (6.0), human carbonic anhydrase (6.5), equine myoglobin (6.8, 7.0), human hemoglobin (7.1), lentil lectin (7.8, 8.0, 8.23), and cytochrome c (9.6).

**Figure 4:**
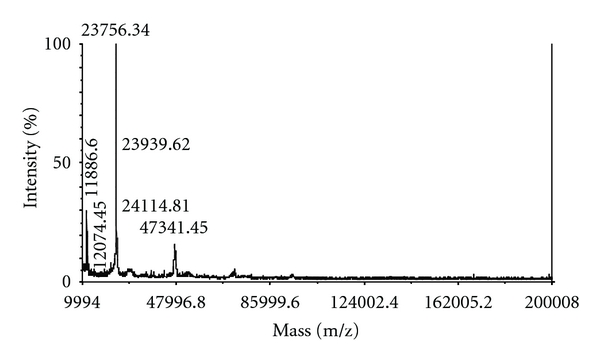
MALDI/TOF-MS spectrum of purified Pacific cod trypsin.

In this study, the stepwise purification of trypsin (TAME esterase activity) from pyloric caeca of the Pacific cod (*G. macrocephalus*) is shown in Table 1. Trypsin was prepared from the pyloric caeca of Pacific cod by affinity chromatography on Benzamidine Sepharose 6B and gel filtration on Superdex 75. The overall purification was increased to 18 fold and the recovery was approximately 64%. In other words, about 50 mg of purified trypsin was obtained from 40 g of acetone powder. It has been reported by Ahsan and Watabe [29] that Benzamidine Sepharose affinity chromatography using benzamidine in a neutral buffer is superior compared to conventional elution procedure with low pH due to the loss of activity in fish trypsin. In this case also, it was similar that affinity chromatography was very effective in removing other protease to the flow-through. In addition, it was considered that the separated protease was trypsin of high purity (Figure 3(a)). Pacific cod trypsin was composed of three isozymes, and their molecular masses were estimated 23,756.34 Da, 23,939.62 Da, and 24114.8 Da by desorption/ionization time-of-flight mass spectroscopy (MALDI/TOF-MS) (Figure 3(b)) and their isoelectric points (pIs), approximately 5.1, 6.0, and 6.2, were found (Figure 4). Moreover, the activity of the purified enzyme did not deteriorate after lyophilization (data not shown).

### 3.2. Enzymatic Properties of Pacific Cod Trypsin

Pacific cod trypsin had the optimum pH of 8.0 and optimum temperature of 50°C for hydrolysis of TAME (Table 2). The enzyme was unstable at above 40°C and at acidic pH, and its thermal stability was highly calcium dependent (Table 2). These properties showed high similarity to other frigid-zone fish trypsins [5]. 

Table 3 shows the results obtained from kinetic measurements using two synthetic substrates (TAME and BAPA). The kinetic parameters of the Pacific cod trypsin were compared with those of bovine trypsin. Pacific cod trypsin hydrolyzed ester bonds at a faster rate than the amide bond. This tendency was also observed for bovine trypsin and for trypsins from fishes [2, 30] and crustaceans [31, 32]. Pacific cod trypsin showed a moderate rate of amide hydrolysis. Kinetic constants of BAPA and TAME hydrolysis by Pacific cod and bovine trypsins were compared at 30°C (Table 3). For BAPA hydrolysis, turnover number (*k*_cat_) of Pacific cod trypsin was 2.2 times faster than that of bovine trypsin, and *k*_*m*_ value of Pacific cod trypsin was considerably lower than bovine enzyme. So, catalytic efficiency for amide hydrolysis expressed as *k*_cat_/*k*_*m*_ of Pacific cod trypsin was 35 times efficient than that of bovine trypsin. For TAME hydrolysis, Pacific cod trypsin also showed faster *k*_cat_ and much lower *k*_*m*_ value than those of bovine counterpart and, consequently, *k*_cat_/*k*_*m*_ of Pacific cod trypsin was about 17 times higher than that of bovine trypsin. Similar to other enzyme and protein, the industrial application for food, cosmetic, and pharmaceutical of mammalian pancreatic trypsin has recently been limited by the outbreak of bovine spongiform encephalopathy (BSE) or religious tradition. So, it is hoped that the new sources of trypsin will be developed. However, there has been little basic research for trypsin from marine organisms comparing with mammalian counterparts. The above results suggest that Pacific cod trypsin can be utilized as alternative enzyme of mammalian for practical use.

In addition, the kinetics of Pacific cod trypsin hydrolysis of the inverse substrate, *N*-Boc-L-Ala-O*p*Am (**5a** in Figure 1) was analyzed as representative examples of the series of compounds. As shown in Table 3, similar results were obtained for* N*-Boc-L-Ala-O*p*Am (**5a**). This result suggests that Pacific cod trypsin may be an efficient catalyst for peptide synthesis.

### 3.3. Pacific Cod Trypsin-Catalyzed Peptide Synthesis

Previously, we reported the behavior of trypsin for the same inverse substrate was different by the origin of enzyme [18, 19] and the yield of coupling reaction with same enzyme was different by the type of inverse substrate [19, 33]. In this paper, twelve types inverse substrates were tested owing to selection of the most efficient substrate in the first using of Pacific cod trypsin for enzymatic peptide synthesis. The reaction conditions for Pacific cod trypsin-catalyzed coupling reaction used effective same condition for multifarious inverse substrates by bovine trypsin as described in the methods [18]. The results of the Pacific cod trypsin-catalyzed peptide coupling reaction are summarized in Table 4. In general, Pacific cod trypsin is a moderately effective catalyst for the synthesis of the peptides (entry 1–24 in Table 4), toward all inverse substrates derived from L-Ala (**1a**–**12a**) and D-Ala (**1b**–**12b**).

In any event, all inverse substrates afforded the coupling product more or less regardless of their structures. The most effective substrate tested in this study was *N*-Boc-alanine *p*-guanidinophenyl esters. They (**6a** and **6b**) afforded the corresponded *N*-Boc-L-Ala-L-Ala-*p*NA (entry 6 in Table 4) and *N*-Boc-D-Ala-L-Ala-*p*NA (entry 18 in Table 4) in 60% and 75% yield, respectively. In the enzymatic peptide synthesis, secondary hydrolysis of the resulting peptide is unavoidable to some extent. This serious problem can be largely overcome by the use of inverse substrates since the resulting peptide is much less specific to the enzyme than the substrate—“preferential coupling to hydrolysis.”

It appears indeed that secondary hydrolysis of the coupling product was negligible in our enzymatic procedure, since a separate experiment incubating the coupling product for 74 h resulted in no detectable change. In this study, it was clarified that Pacific cod trypsin, as well as chum salmon trypsin [33], is suitable for peptide synthesis. However, a thermal stability of fish trypsin is lower than mammalian counterpart, and this property would prevent a massive and continual application of them in industrial peptide synthesis. Now, we are investigating the optimization of trypsin-catalyzed condensation, for example, pH, organic solvent, and concentration of acyl acceptor. We are also trying the immobilization of Pacific cod trypsin to improve its stability for its practical use [34].

## Figures and Tables

**Table 1:** Purification of trypsin from Pacific cod.

Purification stages	Protein (mg)	Total activity (U)	Specific activity (U/mg)	Purity (fold)	Yield (%)
Activated crude extract	503	5,281	10.5	1.0	100
80% (NH_4_)_2_SO_4 _ fraction	424	5,217	12.3	1.2	99
Gel filtration	17.8	3,400	191	18	64

**Table 2:** Enzymatic properties of Pacific cod trypsin.

Enzymatic properties	Pacific cod trypsin
Optimum pH	pH 8.0
Optimum temperature	50°C
pH stability	pH 7.0–10.0
Thermal stability	<40°C

**Table 3:** Kinetic parameters for Pacific cod trypsin-catalyzed hydrolysis.

Substrate	Parameter	PcT	BT	PcT/BT
BAPA	*K*_*m*_ (mM)	0.037	0.57	0.063
	*K*_cat_ (sec^−1^)	3.7	1.7	2.2
	*K*_cat_/*K*_*m*_ (mM^−1^s^−1^)	100	2.9	35

TAME	*K*_*m*_ (mM)	0.0015	0.021	0.074
	*K*_cat_ (sec^−1^)	108	86	1.3
	*K*_cat_/*K*_*m*_ (mM^−1^s^−1^)	71,206	4,160	17

*N*-Boc-L-Ala-O*p*Am (**4a**)	*K*_*m*_ (mM)	0.0015	0.021	0.071
	*K*_cat_ (sec^−1^)	2.0	1.2	1.6
	*K*_cat_/*K*_*m*_ (mM^−1^s^−1^)	1,449	619	2.3

PcT, Pacific cod trypsin; BT, Bovine trypsin.

**Table 4:** Yield of Pacific cod trypsin-catalyzed peptide synthesis.

Entry no.	Acyl donor (no.)	Reaction time (hr)	Product (No.)	Yield (%)
1	*N*-Boc-L-Ala-O*m*Am (**1a**)	48	* N*-Boc-L-Ala-L-Ala-*p*NA (**13a**)	44
2	*N*-Boc-L-Ala-O*m*Gu (**2a**)	24	* N*-Boc-L-Ala-L-Ala-*p*NA (**13a**)	37
3	*N*-Boc-L-Ala-O*m*AM (**3a**)	24	* N*-Boc-L-Ala-L-Ala-*p*NA (**13a**)	55
4	*N*-Boc-L-Ala-O*m*GM (**4a**)	12	* N*-Boc-L-Ala-L-Ala-*p*NA (**13a**)	51
5	*N*-Boc-L-Ala-O*p*Am (**5a**)	0.2	* N*-Boc-L-Ala-L-Ala-*p*NA (**13a**)	58
6	*N*-Boc-L-Ala-O*p*Gu (**6a**)	0.2	* N*-Boc-L-Ala-L-Ala-*p*NA (**13a**)	60
7	*N*-Boc-L-Ala-O*p*AM (**7a)**	5	* N*-Boc-L-Ala-L-Ala-*p*NA (**13a**)	59
8	*N*-Boc-L-Ala-O*p*GM (**8a**)	5	* N*-Boc-L-Ala-L-Ala-*p*NA (**13a**)	59
9	*N*-Boc-L-Ala-O(1-4)GN (**9a**)	5	* N*-Boc-L-Ala-L-Ala-*p*NA (**13a**)	56
10	*N*-Boc-L-Ala-O(1-5)GN (**10a**)	5	* N*-Boc-L-Ala-L-Ala-*p*NA (**13a**)	55
11	*N*-Boc-L-Ala-O(1-6)GN (**11a**)	24	* N*-Boc-L-Ala-L-Ala-*p*NA (**13a**)	52
12	*N*-Boc-L-Ala-O(2-5)GN (**12a**)	50	* N*-Boc-L-Ala-L-Ala-*p*NA (**13a**)	59
13	*N*-Boc-D-Ala-O*m*Am (**1b**)	74	* N*-Boc-D-Ala-L-Ala-*p*NA (**13b**)	12
14	*N*-Boc-D-Ala-O*m*Gm (**2b**)	74	* N*-Boc-D-Ala-L-Ala-*p*NA (**13b**)	52
15	*N* ^*α*^-Boc-D-Ala-O*m*AM (**3b**)	74	* N*-Boc-D-Ala-L-Ala-*p*NA (**13b**)	38
16	*N*-Boc-D-Ala-O*m*GM (**4b**)	74	* N*-Boc-D-Ala-L-Ala-*p*NA (**13b**)	58
17	*N*-Boc-D-Ala-O*p*Am (**5b**)	0.2	* N*-Boc-D-Ala-L-Ala-*p*NA (**13b**)	70
18	*N*-Boc-D-Ala-O*p*Gu (**6b**)	0.2	* N*-Boc-D-Ala-L-Ala-*p*NA (**13b**)	75
19	*N*-Boc-D-Ala-O*p*AM (**7b**)	8	* N*-Boc-D-Ala-L-Ala-*p*NA (**13b**)	76
20	*N*-Boc-D-Ala-O*p*GM (**8b**)	48	* N*-Boc-D-Ala-L-Ala-*p*NA (**13b**)	74
21	*N*-Boc-D-Ala-O(1-4)GN (**9b**)	5	* N*-Boc-D-Ala-L-Ala-*p*NA (**13b**)	57
22	*N*-Boc-D-Ala-O(1-5)GN (**10b**)	97	* N*-Boc-D-Ala-L-Ala-*p*NA (**13b**)	61
23	*N*-Boc-D-Ala-O(1-6)GN (**11b**)	97	* N*-Boc-D-Ala-L-Ala-*p*NA (**13b**)	33
24	*N*-Boc-D-Ala-O(2-5)GN (**12b**)	74	* N*-Boc-D-Ala-L-Ala-*p*NA (**13b**)	23

1 mM acyl donor, 20 mM acyl acceptor, 10 *μ*M PcT, respectively, in MOPS (50 mM, pH 8.0, containing 20 mM CaCl_2_) : DMSO = 1 : 1, at 25°C.

## References

[1] Amiza MA, Apenten RKO (1994). Thermal inactivation parameters for alkaline proteinases from North Sea cod (*Gadus morhua*) and bovine alpha-chymotrypsin. *Journal of the Science of Food and Agriculture*.

[2] Ásgeirsson B, Fox JW, Bjarnason JB (1989). Purification and characterization of trypsin from the poikilotherm *Gadus morhua*. *European Journal of Biochemistry*.

[3] Kristjansson MM (1991). Purification and characterization of trypsin from the pyloric caeca of rainbow trout (*Oncorhynchus mykiss*). *Journal of Agricultural Food and Chemistry*.

[4] Simpson BK, Haard NF, Knorr D (1987). Cold-adapted enzymes from fish. *Food Biotechnology*.

[5] Fuchise T, Kishimura H, Sekizaki H (2009). Purification and characteristics of trypsins from cold-zone fish, Pacific cod (*Gadus macrocephalus*) and saffron cod (*Eleginus gracilis*). *Food Chemistry*.

[6] Tsuzuki H, Oka T, Morihara K (1980). Coupling between Cbz-Arg-OH and leu-X catalyzed by trypsin and papain. *Journal of Biochemistry*.

[7] Nakatsuka T, Sasaki T, Kaiser ET (1987). Peptide segment coupling catalyzed by the semisynthetic enzyme thiolsubtilisin. *Journal of the American Chemical Society*.

[8] Wong CH (1989). Enzymatic catalysts in organic synthesis. *Science*.

[9] Schellenberger V, Jakubke HD (1991). Protease-catalyzed kinetically controlled peptide synthesis. *Angewandte Chemie*.

[10] Gill I, López-Fandiño R, Jorba X, Vulfson EN (1996). Biologically active peptides and enzymatic approaches to their production. *Enzyme and Microbial Technology*.

[11] Bordusa F (2002). Proteases in organic synthesis. *Chemical Reviews*.

[12] Kumar D, Bhalla TC (2005). Microbial proteases in peptide synthesis: approaches and applications. *Applied Microbiology and Biotechnology*.

[13] Itoh K, Sekizaki H, Toyota E, Fujiwara N, Tanizawa K (1996). Application of inverse substrates to trypsin-catalyzed peptide synthesis. *Bioorganic Chemistry*.

[14] Itoh K, Sekizaki H, Toyota E, Tanizawa K (1995). Synthesis and properties of *N*-(*tert*-butyloxycarbonyl)peptide *p*- guanidinophenyl esters as trypsin substrates. *Chemical and Pharmaceutical Bulletin*.

[15] Sekizaki H, Itoh K, Toyota E, Tanizawa K (1996). Synthesis and tryptic hydrolysis of *p*-guanidinophenyl esters derived from amino acids and peptides. *Chemical and Pharmaceutical Bulletin*.

[16] Tanizawa K, Kasaba Y, Kanaoka Y (1977). “Inverse substrates” for trypsin. Efficient enzymatic hydrolysis of certain esters with a cationic center in the leaving group. *Journal of the American Chemical Society*.

[17] Sekizaki H, Itoh K, Toyota E, Tanizawa K (1996). Trypsin-catalyzed peptide synthesis with various *p*-guanidinophenyl esters as acyl donors. *Chemical and Pharmaceutical Bulletin*.

[18] Sekizaki H, Itoh K, Toyota E, Tanizawa K (1998). Enzymatic peptide synthesis with *p*-guanidinophenyl and *p*- (guanidinomethyl)phenyl esters as acyl donors. *Chemical and Pharmaceutical Bulletin*.

[19] Sekizaki H, Toyota E, Fuchise T, Zhou S, Noguchi Y, Horita K (2008). Application of several types of substrates to ficin-catalyzed peptide synthesis. *Amino Acids*.

[20] Sekizaki H, Itoh K, Toyota E, Tanizawa K (1999). Trypsin-catalyzed peptide synthesis with *m*-guanidinophenyl and *m*-(guanidinomethyl)phenyl esters as acyl donor component. *Amino Acids*.

[21] Sekizaki H, Itoh K, Toyota E, Tanizawa K (1999). The structural requirements for an inverse substrate for enzymatic peptide synthesis: position isomers of guanidinonaphthyl esters as the acyl donor component. *Chemical and Pharmaceutical Bulletin*.

[22] Sekizaki H, Itoh K, Shibuya A, Toyota E, Tanizawa K (2007). A facile synthesis of *p*- and *m*-(amidinomethyl)phenyl esters derived from amino acid and tryptic hydrolysis of these synthetic inverse substrates. *Chemical and Pharmaceutical Bulletin*.

[23] Bradford MM (1976). A rapid and sensitive method for the quantitation of microgram quantities of protein utilizing the principle of protein dye binding. *Analytical Biochemistry*.

[24] Hummel BCW (1959). A modified spectrophotometric determination of chymotrypsin, trypsin, and thrombin. *Canadian Journal of Biochemistry and Physiology*.

[25] Satvinder KS, Maxwell TH (1990). Microplate reader-based kinetic determination of *α*-amylase activity: application to quantitation of secretion from rat parotid acini. *Analytical Biochemistry*.

[26] Chase T, Shaw E (1967). *p*-Nitrophenyl-*p*′-guanidinobenzoate HCl: a new active site titrant for trypsin. *Biochemical and Biophysical Research Communications*.

[27] Erlanger BF, Kokowsky N, Cohen W (1961). The preparation and properties of two new chromogenic substrates of trypsin. *Archives of Biochemistry and Biophysics*.

[28] Laemmli UK (1970). Cleavage of structural proteins during the assembly of the head of bacteriophage T4. *Nature*.

[29] Ahsan MN, Watabe S (2001). Kinetic and structural properties of two isoforms of trypsin isolated from the viscera of japanese anchovy, engraulis japonicus. *Journal of Protein Chemistry*.

[30] Simpson BK, Smith JP, Yaylayan V, Haard NF (1989). Kinetic and thermodynamic characteristics of a digestive protease from Atlantic cod, *Gadus morhua*. *Journal of Food Biochemistry*.

[31] DeVillez E, Buschlen K (1967). Survey of a tryptic digestive enzyme in various species of crustacea. *Comparative Biochemistry and Physiology*.

[32] Osnes KK, Mohr V (1985). On the purification and characterization of three anionic, serine-type peptide hydrolases from antarctic krill, *Euphausia Superba*. *Comparative Biochemistry and Physiology B*.

[33] Sekizaki H, Itoh K, Toyota E, Tanizawa K (2001). Chum salmon trypsin-catalyzed preferential formation of peptides containing D-amino acid. *Amino Acids*.

[34] Mateo C, Palomo JM, Fernandez-Lorente G, Guisan JM, Fernandez-Lafuente R (2007). Improvement of enzyme activity, stability and selectivity via immobilization techniques. *Enzyme and Microbial Technology*.

